# Deguelin suppresses angiogenesis in human hepatocellular carcinoma by targeting HGF-c-Met pathway

**DOI:** 10.18632/oncotarget.22077

**Published:** 2017-10-26

**Authors:** Ming Li, Xinfang Yu, Wei Li, Ting Liu, Gang Deng, Wenbin Liu, Haidan Liu, Feng Gao

**Affiliations:** ^1^ Department of Ultrasonography, The Third Xiangya Hospital of Central South University, Changsha, Hunan 410013, P.R. China; ^2^ State Key Laboratory of Powder Metallurgy, Central South University, Changsha, Hunan 410006, P.R. China; ^3^ Clinical Center for Gene Diagnosis and Therapy, The Second Xiangya Hospital of Central South University, Changsha, Hunan 410011, P.R. China; ^4^ School of Stomatology, Hunan University of Chinese Medicine, Changsha, Hunan 410208, P.R. China; ^5^ Changsha Stomatological Hospital, Changsha, Hunan 410004, P.R. China; ^6^ Department of Cancer Biology, Lerner Research Institute, Cleveland Clinic, Cleveland, Ohio 44195, USA; ^7^ Department of Radiology, The Third Xiangya Hospital of Central South University, Changsha, Hunan 410013, P.R. China; ^8^ Department of Hepatobiliary and Pancreatic Surgery, The Third Xiangya Hospital of Central South University, Changsha, Hunan 410013, P.R. China; ^9^ Hunan Cancer Hospital and The Affiliated Cancer Hospital of Xiangya School of Medicine, Central South University, Changsha, Hunan 410013, P.R. China

**Keywords:** hepatocellular carcinoma, deguelin, angiogenesis, VEGF, c-Met

## Abstract

Angiogenesis plays a crucial role in the development of human hepatocellular carcinoma (HCC). In the present study, we found a natural compound, deguelin, has a profound anti-angiogenesis effect on HCC. Deguelin suppressed vascular endothelial growth factor (VEGF)-induced human umbilical vascular endothelial cells (HUVECs) proliferation, migration, invasion, and capillary-like tube formation *in vitro* and reduced tumor angiogenesis *in vivo*. We discovered that VEGF receptor-mediated signal transduction cascades in HUVECs were inhibited by deguelin. Deguelin decreased the autocrine of VEGF in HCC cells in a time- and dose-dependent manner. Additionally, deguelin suppressed HGF-induced activation of the c-Met signaling pathway. Knocking down c-Met or inhibition of c-Met activation impaired HGF-mediated VEGF production. Importantly, we produced patient-derived hepatocellular carcinoma xenografts to evaluate the therapeutic effect of deguelin *in vivo*. Taken together, these results indicate that deguelin could inhibit HCC through suppression of angiogenesis on vascular endothelial cells and reduction of proangiogenic factors in cancer cells.

## INTRODUCTION

Tumor angiogenesis, the development of new blood vessels from the existing vasculature, is considered to play an essential role in malignant neoplasia development [1]. It is estimated that over 90% cancer deaths that occur are due to angiogenesis, invasion, and distant metastasis of cancer to vital organs [2]. The proliferation and migration of endothelial cells in response to chemotactic agents, such as vascular endothelial growth factor (VEGF), are considered a key step in the initiation of angiogenesis [3]. VEGF exerts its biological effects by binding to its receptor tyrosine kinases, VEGFR1, VEGFR2, and VEGFR3. VEGFR2 plays an important role in mediating the mitogenesis and permeability of endothelial cells [4]. Activation of VEGFR2 leads to the phosphorylation of various downstream signal transduction pathways, including extracellular signal-regulated kinases (ERK), protein kinase C, Src family kinase, focal adhesion kinase, and PI3-K/AKT pathway [4, 5]. VEGFR2 targeting therapies or angiogenesis blockade has been shown to be an effective strategy in inhibiting tumor growth and metastasis [6–8].

Hepatocellular carcinoma (HCC), the most common primary liver tumor, is one of the most aggressive and lethal malignancies. Overall, the 5-year survival rate for patients with HCC of any stage is about 15%. Resistant to systemic therapies and often recurs even after aggressive local therapies are the main problems in HCC treatment [9, 10]. Understanding the molecular mechanisms of the complex multistep process of HCC could facilitate the development of preventive measures, early diagnostic methods, and better treatments. The formation of new blood vessels plays a key role in HCC, which is responsible for the rapid recurrence and poor survival. New vessel formation with abnormal structure and function leads to an abnormal tumor microenvironment characterized by low oxygen tension. The liver is perfused by both arterial and venous blood and the resulting abnormal microenvironment selects for more aggressive malignancies [11, 12]. Indeed, a large number of antiangiogenic agents, such as bevacizumab [13], Sorafenib [14, 15] and Sunitinib [16, 17], are currently being tested for the treatment of advanced-stage HCC.

Deguelin, a natural product isolated from several plant species such as Mundulea sericea, has been demonstrated that it exerted anti-proliferation activities and/or induce apoptosis in a panel of human cancers, such as lung cancer [18–20], colon cancer [21], pancreatic Cancer [22], prostate cancer [23] and breast cancer [24, 25]. Mechanism investigation manifested that deguelin treatment resulted in cell cycle arrest [26], induction of apoptosis [27], inhibition the activities of NF-κB [28] and PI3K/Akt signaling pathways [18]. Nonetheless, the anti-tumor activity of deguelin in hepatocellular carcinoma, as well as the effect on tumor angiogenesis has not yet been fully investigated.

In this study, we have identified deguelin as a novel inhibitor of tumor angiogenesis and characterized its underlying molecular mechanisms. The anti-angiogenic properties of deguelin were evaluated *in vitro* using HUVECs proliferation, migration, and tubular formation assays and *in vivo* by Matrigel plug assay. Moreover, we demonstrated that deguelin suppressed VEGF secretion in an HGF/c-MET axis dependent manner in HCC cells. Most importantly, we evaluated the possible clinical use of deguelin by investigating its therapeutic effects in patient-derived xenografts (PDXs) of primary human hepatocellular carcinoma.

## RESULTS

### Deguelin inhibits hepatocellular carcinoma cells growth *in vitro* and *in vivo*

**Figure 1:**
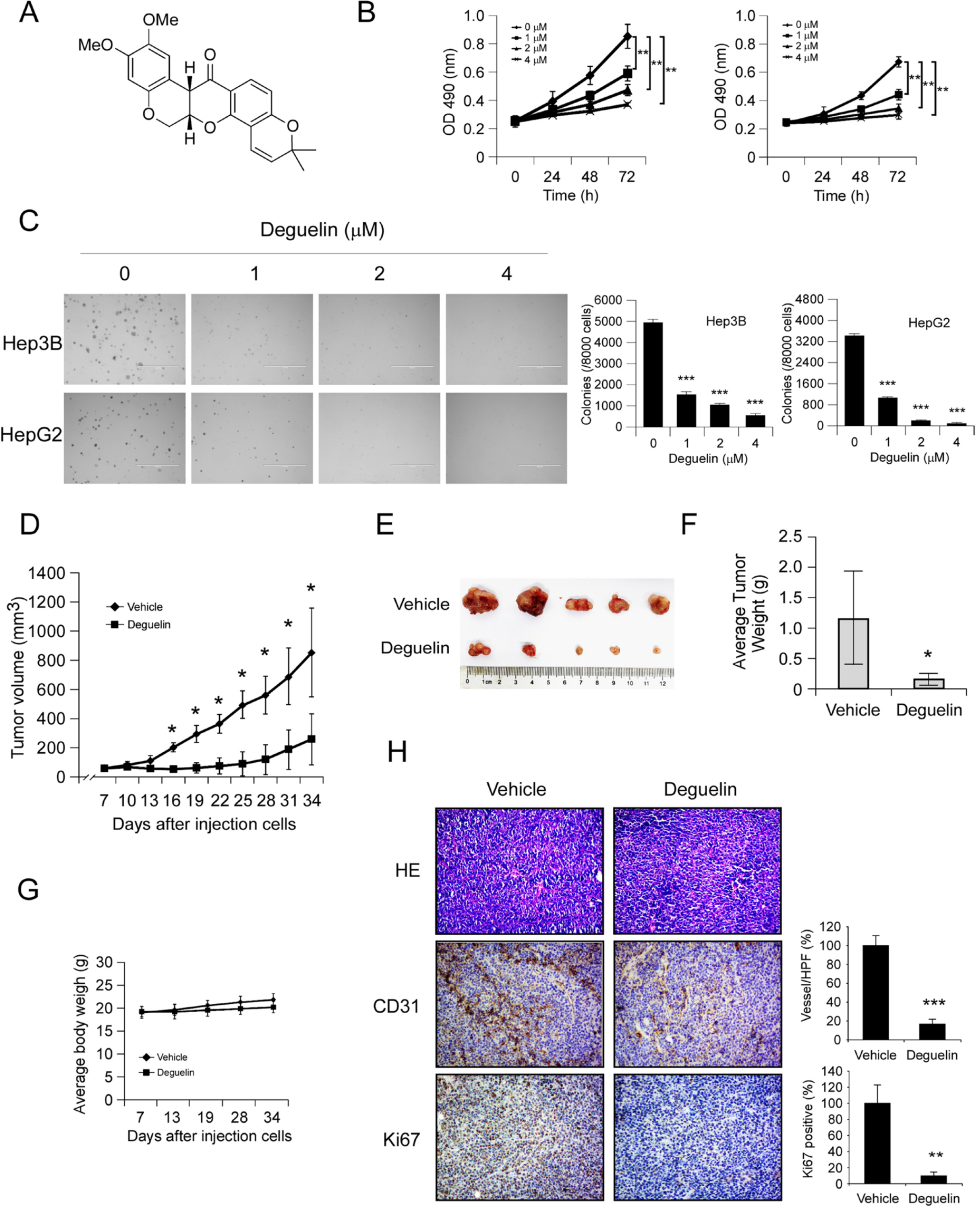
Deguelin inhibits hepatocellular carcinoma cells growth *in vitro* and *in vivo* (**A**) Chemical structure of deguelin. (**B**) Deguelin decreases cell growth. Hep3B (left) and HepG2 (right) cells were treated or not treated with deguelin as indicated, MTS assay was performed as described in Materials and Methods. The asterisk (^**^) indicates a significant (*p* < 0.01) decrease in cell proliferation by deguelin-treated cells. (**C**) Deguelin attenuates Hep3B and HepG2 anchorage-independent cell growth. Soft agar assay was performed as described in Materials and Methods. The asterisk (^***^) indicates a significant (*p* < 0.001) decrease in colony formation by deguelin-treated cells. (**D**–**G**) Deguelin inhibits tumor growth *in vivo*. (D) Tumor growth curve, (E) photograph of tumors in the vehicle and deguelin-treated group, (F) average tumor weight, (G) average body weight of mice. Data are represented as means ± SD of each group. The asterisk (^*^) indicates a significant difference (*p* < 0.05) compared with the deguelin-treated group. (**H**) Immunohistochemical examination of CD31 and Ki67 in tumor sections. Left panel, a representative photograph of tumor tissue per group (200×); right panel, the expression of indicated protein in per group was quantified, the asterisks (^***^p < 0.001) indicates a significant difference.

First, we investigated the activity of deguelin (Figure 1A) against hepatocellular carcinoma cells proliferation in Hep3B, HepG2 and MHCC97-H cells. MTS data showed that deguelin significantly decreased the anchorage-dependent growth of Hep3B, HpeG2 (Figure 1B), and MHCC97-H cells (Supplementary Figure 1A) in a dose-dependent manner. Moreover, Treatment with deguelin dramatically inhibited anchorage-independent cell growth in Hep3B, HepG2 (Figure 1C) and MHCC97-H cells (Supplementary Figure 1B). In order to further confirm the antitumor activity of deguelin, the potency of deguelin against HCC cell growth was investigated *in vivo*. As shown in Figure 1D–1F, tumor growth in the deguelin-treated group was delayed in contrast with the vehicle group. The average tumor volume of vehicle treated group had reached about 800 mm^3^, but in deguelin treated group, the average tumor volume was only around 300 mm^3^. Meanwhile, during the treatment period, deguelin did not affect the body weight obviously (Figure 1G). Our results found that deguelin can’t significantly induce the up/down-regulation of the AST, ALT and BUN, which indicated that the deguelin has no obvious hematotoxicity (Supplementary Figure 2). Immunohistochemistry analysis revealed that the positive staining of Ki67 in tumor tissue was substantially decreased after deguelin treatment, which confirmed the anti-proliferation activity of deguelin on HCC *in vivo*. Interestingly, we found that CD31 staining for newly formed blood vessels in deguelin treated group was decreased by 60% compared to vehicle-treated group (Figure 1H), which indicated that the anti-tumor effect of deguelin may partly dependent on the inhibition of angiogenesis.

### Deguelin decreases cell proliferation in HUVECs

**Figure 2:**
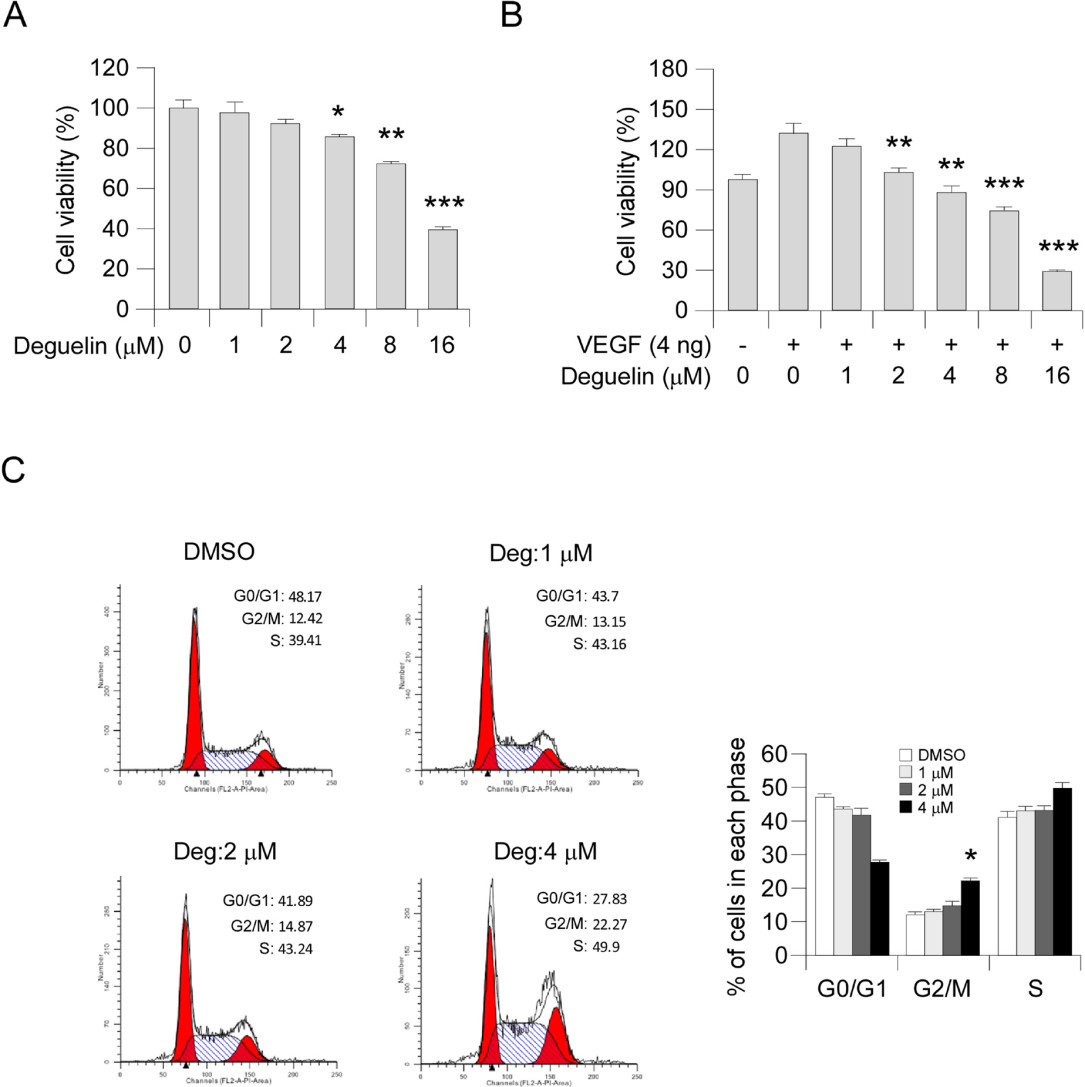
Deguelin inhibits the proliferation of HUVECs (**A**) Effects of deguelin on HUVECs proliferation under normal culture condition. HUVECs (2 × 10^4^ per well) were treated with different concentrations of deguelin for 24 h, cell proliferation was tested by MTS assay. The asterisk indicates a significant (^*^p < 0.05, ^**^p < 0.01, ^***^p < 0.001,) decrease in cell proliferation by deguelin-treated cells. (**B**) Deguelin inhibits VEGF-induced cell proliferation in a dose-dependent manner. HUVECs (2 × 10^4^ per well) were starved with 0.5% FBS medium and then treated with or without VEGF (4 ng/mL) and different concentrations of deguelin for 24 h. Cell proliferation was quantified by MTS assay. The asterisk indicates a significant (^**^p < 0.01, ^***^p < 0.001,) decrease in cell proliferation by deguelin-treated cells. (**C**) Deguelin-induced cell cycle G2/M arrest. HUVECs were treated as described in Materials and Methods, and flow cytometry analysis was used to analyze deguelin-induced cell cycle arrest. The asterisk indicates a significant (^*^p < 0.05) increase in G2/M phase after deguelin treatment.

To assess the antiangiogenic property of deguelin *in vitro*, we examined the inhibitory effects of deguelin on cell proliferation of HUVECs. Our results showed that deguelin had no obvious effect on cell proliferation at a low concentration (1–2 μM) but substantially inhibited HUVECs growth after the concentration reached >4 μM (Figure 2A). Moreover, deguelin significantly suppressed VEGF-induced endothelial cell proliferation from 2 μM (Figure 2B), indicating that deguelin is more effective in angiogenesis disease condition. To examine whether deguelin-mediated HUVECs suppression was related to cell cycle progression deregulation, we performed FACS analysis. The results revealed that deguelin-induced accumulation of cells at G2/M phase (Figure 2C). These data suggested that deguelin could arrest HUVECs cell cycle progression *in vitro*.

### Deguelin suppresses VEGF-induced migration, invasion, capillary tube formation of HUVECs *in vitro* and VEGF-induced angiogenesis *in vivo*

**Figure 3:**
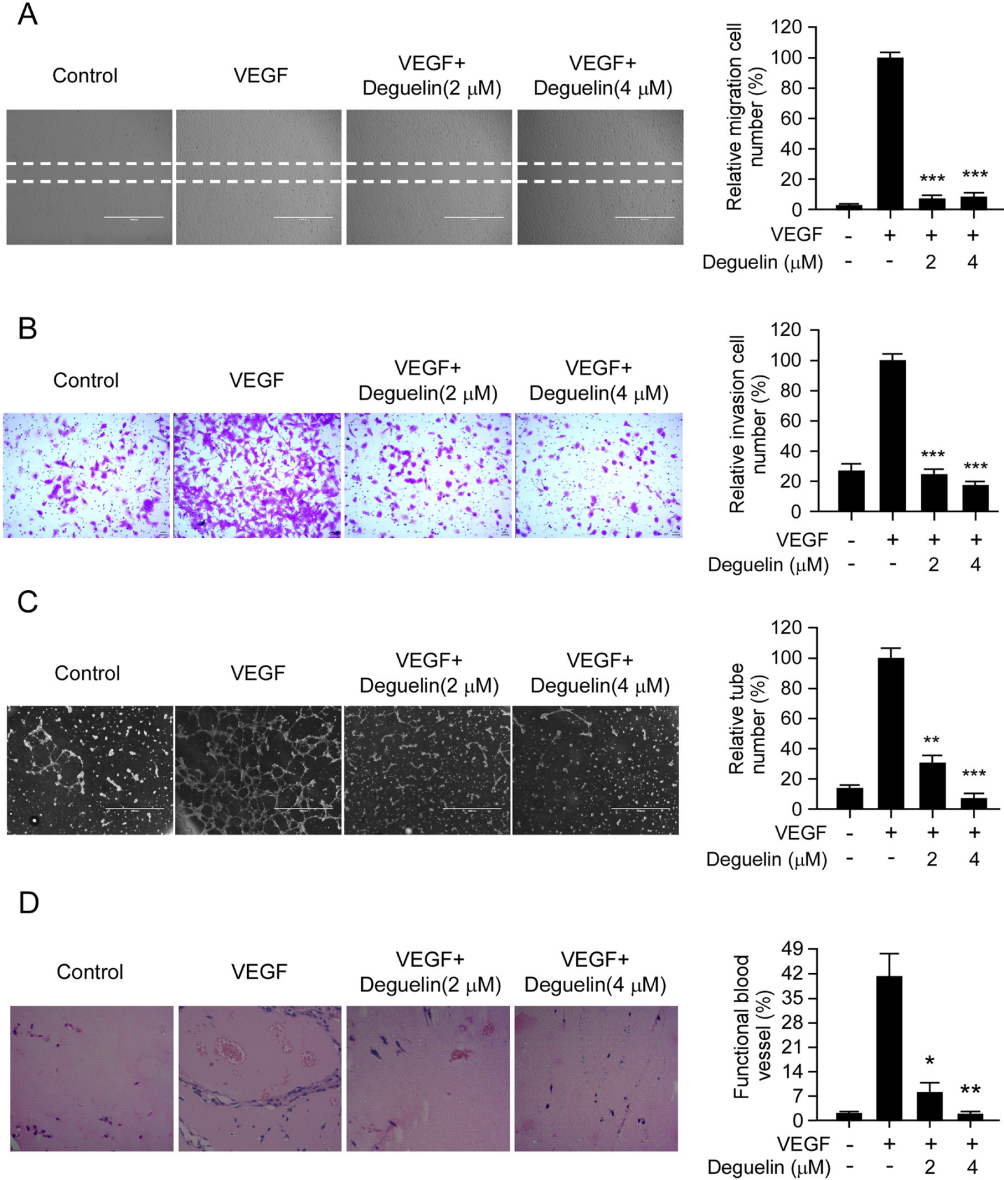
Deguelin suppresses VEGF-induced migration, invasion, capillary tube formation of HUVECs *in vitro* and VEGF-induced angiogenesis *in vivo* **(A**) HUVECs were allowed to grow to full confluence in six-well plates and starved in 0.5% FBS medium. Cells were scratched with a pipette tip, then treated with different concentrations of deguelin and 10 ng/mL VEGF in M199 supplemented with 0.5% FBS as indicated. After 12 h, the migrated cells were quantified by manual counting. (**B**) HUVECs were seeded in the upper chamber of a Transwell slide and treated with different concentrations of deguelin. The bottom chamber was filled with ECGM supplemented with VEGF (10 ng/mL). After 8 h, cells adherent on the lower surface of the filters were fixed and stained. Five representative fields were photographed under a light microscope and counted in triplicate to obtain invasion indices. (**C**) HUVECs were placed on the top of polymerized Matrigel in 24-well plates in the absence or presence of different concentrations of deguelin as indicated. After 8 h, tubular structures were photographed. Tube formation was quantified by manual counting of high power fields (HPFs). (**D**) Matrigel (0.5 mL) containing VEGF (80 ng) and the indicated amounts of deguelin was injected into C57BL/6 mice in the midventral abdominal region (5 mice per group). After 7 days, Matrigel plugs were removed and fixed in formalin. Sections were stained with H&E. The number of vessels in HPF was counted for 5 representative fields in triplicate under a light microscope. Left panel, a representative photograph of each group; right panel, the quantified data of each group. Each experiment was conducted 3 times and the data are expressed as mean values ± S.D. (^*^p < 0.05, ^**^p < 0.01, ^***^p < 0.001 versus VEGF-treated group).

Cell migration is a critical step for the endothelial cell to form blood vessels in angiogenesis. Based on the previous data, we further determined the inhibitory effects of deguelin on the chemotactic motility of endothelial cells using the wound-healing migration assay and transwell assay, respectively. The results showed that deguelin significantly inhibited VEGF-induced HUVECs migration in a dose-dependent manner (Figure 3A). Because directional motility and matrix degradation are crucial for angiogenesis sprouting, we next used the Boyden chamber transwell assay to determine whether deguelin affected the motility of HUVECs. Our results showed that deguelin dramatically reduced cell invasion (Figure 3B). Next, the ability of endothelial cells to form tube-like structures was assessed with an inverted photomicroscope. We found that robust tubular-like structures were formed in the presence of VEGF, but exposure to deguelin dose-dependently down-regulated VEGF-induced tubule formation of HUVECs (Figure 3C). We further performed the mouse matrigel plug assay to analyze how deguelin regulated VEGF-induced angiogenesis *in vivo*. Matrigel and heparin were mixed or not mixed with deguelin (2 µM and 4 µM). The mixture was injected into C57BL/6 mice, and the Matrigel plugs were removed after 7 days. The plugs containing VEGF alone appeared dark red, whereas the color of the Matrigel plugs containing VEGF plus deguelin was substantially paler, indicating less blood vessel formation (data not shown). Hematoxylin and eosin (H&E) staining showed that the plugs containing VEGF alone exhibited functional vasculature formation inside the Matrigel through angiogenesis. In contrast, deguelin dramatically decreased VEGF-induced microvessel number in a dose-dependent manner (Figure 3D). Taken together, these results indicated that deguelin was capable of suppressing VEGF-induced neovessel formation both *in vitro* and *in vivo*.

### Deguelin down-regulates VEGF production in HCC cells and suppresses VEGFR2 signaling pathway in HUVECs

**Figure 4:**
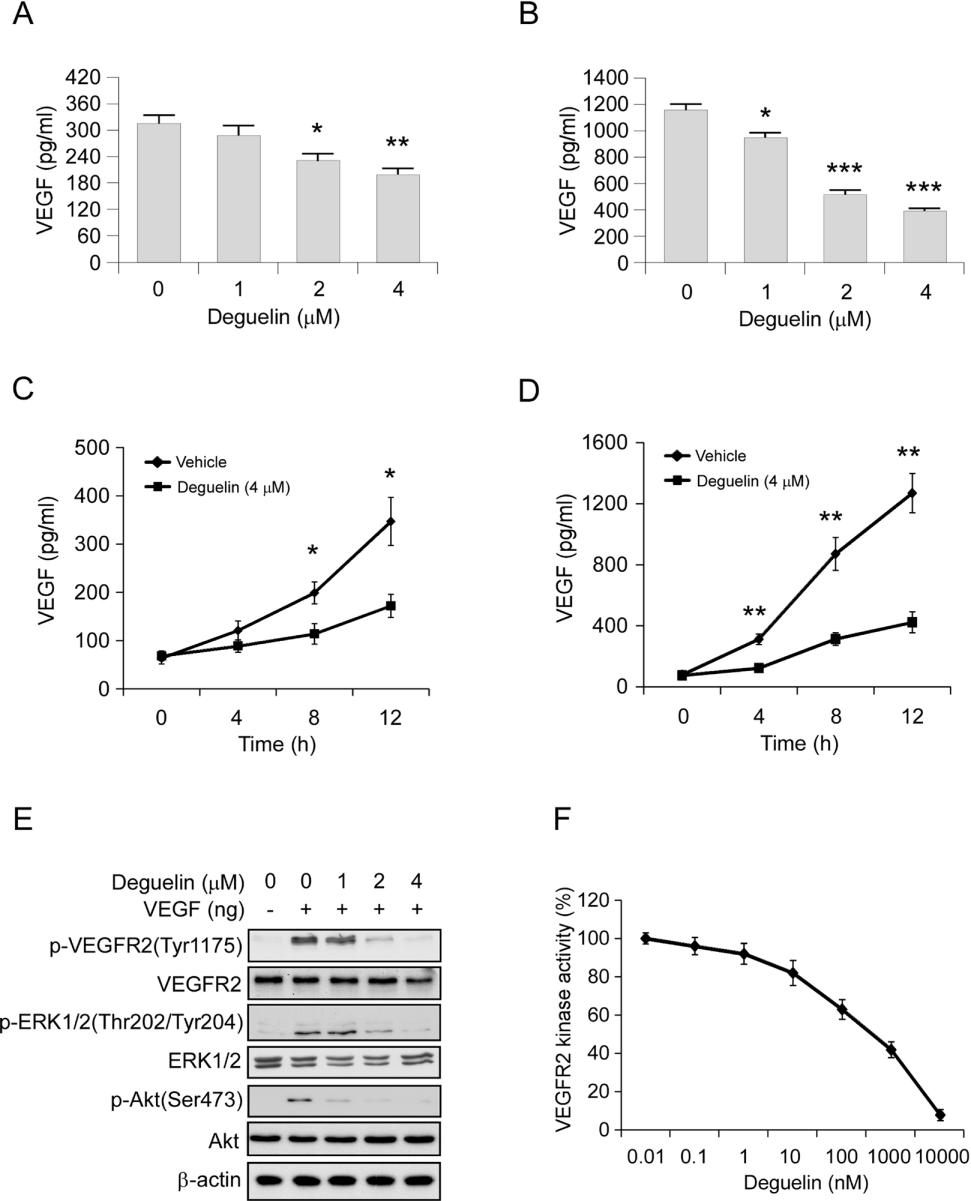
Deguelin inhibits VEGF production in HCC cells and suppresses VEGFR2 signaling pathway in HUVECs **(A**) and (**B**) Hep3B (A) and HepG2 (B) cells were treated with different concentrations of deguelin for 12 h. Cell culture media was harvested and VEGF level was measured by ELISA assay. (**C**) and (**D**) Hep3B (C) and HepG2 (D) cells were treated or not treated with 4 μM deguelin. Cell culture media was harvested at different time points and VEGF level was measured by ELISA assay. (**E**) Deguelin inhibits VEGF-induced VEGFR2 activation in HUVECs. HUVECs were starved in 0.5% FBS medium overnight and treated with various dosages of deguelin for 2 h, the cells were stimulated with VEGF (40 ng/ml) for 30 min. Cell lysates were then subjected to SDS-PAGE followed by Western blotting. β-Actin served as a loading control. (**F**) Inhibition of deguelin on VEGFR2 activation in a specific VEGFR2 inhibition assay. The experiment was conducted 3 times and the data are expressed as mean values ± S.D. (^*^p < 0.05, ^**^p < 0.01, ^***^p < 0.001 versus untreated/vehicle group).

In order to explore the mechanism involved in deguelin-mediated anti-angiogenesis effect, we first determined the autocrine of VEGF in Hep3B and HepG2 cells via ELISA assay. Results showed that the VEGF protein levels in the cell culture medium were significantly decreased in a dose-dependent manner in deguelin treated group, and the HepG2 cell secreted much higher levels of VEGF than the Hep3B cell (Figure 4A, 4B). Moreover, we found that deguelin decreased VEGF production in a time-dependent manner in both Hep3B and HepG2 cells (Figure 4C, 4D). VEGFR2 is the primary receptor in VEGF signaling pathway that regulates endothelial cell proliferation, migration, differentiation, tube formation, and angiogenesis. As shown in Figure 4E, deguelin substantially inhibited VEGF-induced VEGFR2, Akt and ERK1/2 activation (Figure 4E), which suggests that deguelin is a potential inhibitor of VEGFR2. Thus, we further examined the effects of deguelin on the specific activation of VEGFR2 using HTScan VEGFR2 kinase assay kit. We found that deguelin inhibited VEGFR2 activation directly in a dose-dependent manner (Figure 4F). These results suggested that the antiangiogenic property of deguelin may be at least partially dependent on the suppression of VEGF secretion and VEGFR2 activation.

### Deguelin inhibits VEGF production through HGF-cMet signaling pathway

**Figure 5:**
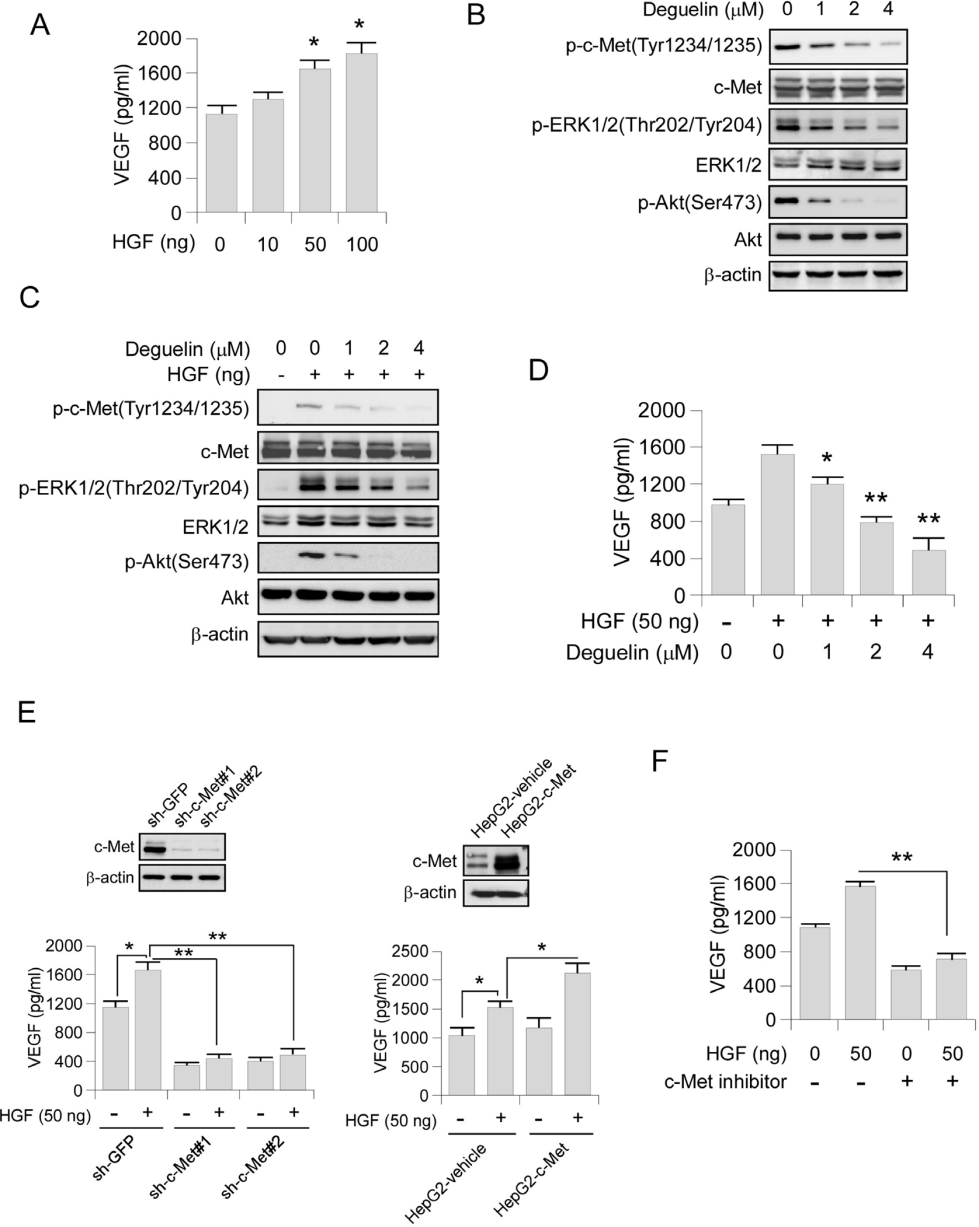
Deguelin inhibits VEGF production through HGF-cMet signaling pathway **(A**) HGF promotes VEGF secretion. HepG2 cells were treated with different concentrations of HGF for 12 h. Cell culture media was harvested and VEGF level was measured by ELISA assay. (**B**) Deguelin inhibits c-Met signaling pathway in HepG2. HepG2 cells were treated with various dosages of deguelin for 24 h, cell lysates were subjected to SDS-PAGE followed by Western blotting as indicated. β-Actin served as a loading control. (**C**) Deguelin inhibits HGF-induced c-Met signaling pathway in HepG2. HepG2 cells were starved in 0.1% FBS medium overnight and treated with various dosages of deguelin for 2 h, the cells were treated with HGF (50 ng/ml) for 30 min. Cell lysates were subjected to SDS-PAGE followed by Western blotting as indicated. β-Actin served as a loading control. (**D**) Deguelin inhibits HGF-induced VEGF secretion. HepG2 cells were pretreated with different concentrations of deguelin for 2 h and followed by HGF treatment for 12 h. Cell culture media was harvested and VEGF level was measured by ELISA assay. (**E**) Knocking down (left) of c-Met impaired HGF-induced VEGF secretion. The c-Met expression was tested by Western blot (top). The sh-GFP and sh-c-Met cells were treated with or without HGF for 12 h, cell culture media was subjected to ELISA assay. Overexpression of c-Met (right) promoted HGF-induced VEGF secretion. The c-Met expression was tested by Western blot (top). The HepG2-vehicle and HepG2-c-Met cells were treated with or without HGF for 12 h, cell culture media was subjected to ELISA assay. (**F**) c-Met inhibitor suppresses HGF-induced VEGF secretion. HepG2 cells were pretreated with or without c-Met inhibitor for 2 h and followed by HGF stimulation for another 12 h, cell culture media was subjected to ELISA assay. Each experiment was conducted 3 times and the data are expressed as mean values ± S.D. (^*^p < 0.05, ^**^p < 0.01 versus untreated/vehicle group).

The HGF-c-Met signaling pathway is a hub in the regulation of malignant progression in HCC. Suppression of c-Met has been reported to inhibit angiogenesis through regulating the expression of angiogenesis factors, such as VEGF [29–31]. Indeed, we found that the secretion of VEGF in HepG2 cells was substantially upregulated with the stimulation of HGF (Figure 5A). Western blot analysis showed that the activation of the key components of c-Met signaling pathway, including phosphorylation of c-Met, Akt and ERK1/2 were decreased in deguelin-treated HepG2 cells (Figure 5B). Consistently, HGF-induced phosphorylation of c-Met, as well as the activation of its downstream signaling, was also inhibited by deguelin in a dose-dependent manner (Figure 5C). Exposure to deguelin dramatically inhibited HGF-induced upregulation of VEGF secretion in HepG2 cells as expected (Figure 5D). To further elucidate the specific role of c-Met in the HGF-induced VEGF secretion, we constructed c-Met stable knocking down HepG2 cells. Our data showed that down-regulation of c-Met expression blocked HGF-increased VEGF production (Figure 5E, left). Overexpression of c-Met in HepG2 cells promotes HGF-induced VEGF secretion (Figure 5E, right). Furthermore, c-Met inhibitor also inhibited HGF-induced VEGF up-regulation (Figure 5F). In order to confirm that deguelin-induced VEGF secretion suppression was only dependent on HGF-c-Met signaling inhibition, we detected the effect of deguelin on other tyrosine kinase receptors, including EGFR and IGF1Rβ. We found that deguelin substantially inhibited EGFR activity (Supplementary Figure 3A) in HepG2 cell. Although EGF treatment activated the EGFR signaling pathway (Supplementary Figure 3B), the VEGF production in HepG2 cells was not promoted significantly (Supplementary Figure 3C). The further experiment showed that the EGFR specific inhibitor, gefitinib, had no obvious effect on VEGF secretion (Supplementary Figure 3D), and knockdown of EGFR in HepG2 cells didn’t induce the decrease of VEGF production (Supplementary Figure 3E). As the data showed in Supplementary Figure 4A, deguelin can’t effectively inhibit the activation of IGF1Rβ. Similarly, we also found that the IGF1R signaling pathway was not essential for deguelin-induced down-regulation of VEGF production, IGF1 can’t dramatically increase the protein level of VEGF (Supplementary Figure 4B, 4C), and inhibited the activity of IGF1Rβ by small-molecule inhibitor or knockdown of IGF1Rβ by shRNA had no significant effect on VEGF secretion (Supplementary Figure 4D, 4E). Collectively, these results indicated that a c-Met activation is a critical event contributing to VEGF production in HCC cells.

### *In vivo* antitumor efficacy of deguelin in human hepatocellular PDX tumor models

**Figure 6:**
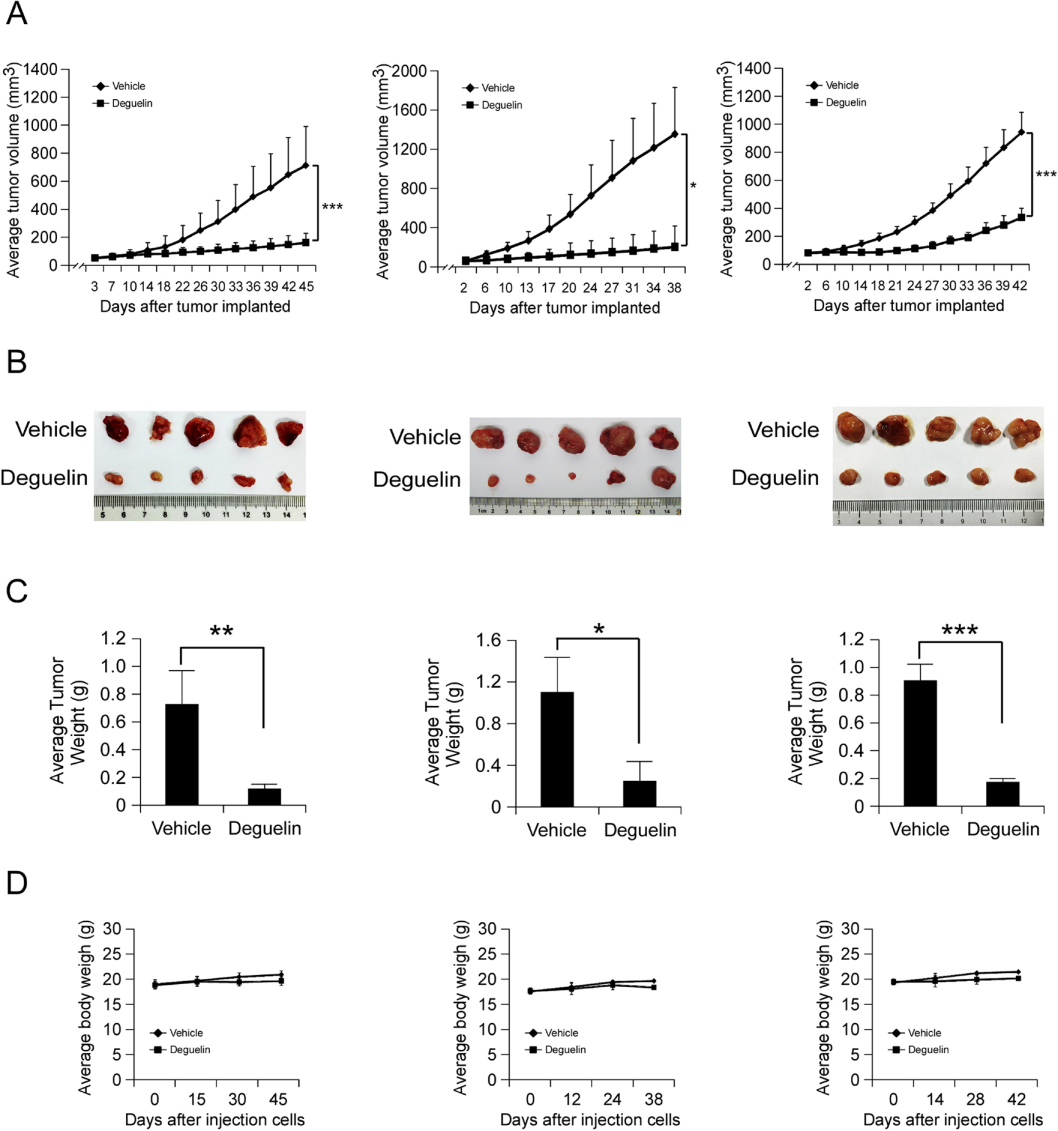
*In vivo* antitumor efficacy of deguelin in human hepatocellular PDX tumor models (**A–C**) Deguelin significantly inhibits tumor growth in a PDX tumor model compared to a vehicle-treated group. B-17 SCID mice each implanted with a different patient’s tumor were divided into 2 groups. Mice implanted with Case 1 (left, *n* = 10), Case 2 (middle, *n* = 10) or Case 3 (right, *n* = 10) were treated every three days by i.p. injection with vehicle or deguelin. (A) Tumor growth curve. (B) Photograph of tumors in the vehicle and deguelin-treated group. (C) Average tumor weight. (**D**) The change of body weight of tumor-bearing mice during the experiment. Data are represented as mean values ± S.D. The asterisks indicate significant differences (^*^p < 0.05, ^**^p < 0.01, ^**^p < 0.001) between deguelin-treated and vehicle-treated group.

To further confirm the antitumor activity of deguelin *in vivo*, we utilized a human hepatocellular PDX tumor model. This model is based on the transfer of primary tumors directly from the human patient into an immune deficient mouse. We selected three tumors having the same cancer grade and stage in order to maintain consistency (Table 1). None of the PDX tumors received chemotherapy before surgery and subsequent implantation. After the original tumor specimen was serially passaged to treatment phase 3 (P3), vehicle or deguelin was administered by intraperitoneal injection. As shown in Figure 6A–6C, deguelin displayed a significant antitumor effect in PDX tumor model. At the endpoint, the average tumor weights of vehicle group and deguelin group in each case were 0.698 ± 0.268/0.09 ± 0.023 g, 1.094 ± 0.329/0.251 ± 0.249 and 0.91 ± 0.108/0.135 ± 0.011, respectively. Notably, no significant differences in body weights occurred among the three xenograft groups treated or not treated with deguelin (Figure 6D). All these *in vivo* data demonstrated that deguelin treatment provides positive effects on human hepatocellular carcinoma.

**Table 1:** Clinical characteristics of the origin used in PDX tumor models

Model ID	Gender	Age (yrs)	Source	Histology	Cancer grade	Cancer stage	Prior chemo
**Case 1**	Male	51	Primary	HCC	IIa	T2N0M0	No
**Case 2**	Male	57	Primary	HCC	IIa	T2N0M0	No
**Case 3**	Male	59	Primary	HCC	IIa	T2N0M0	No

Abbreviations: HCC: hepatocellular carcinoma; T: tumor; N: lymph nodes; M: metastasis.

## DISCUSSION

In the present study, we demonstrated that deguelin exerted a substantial anti-angiogenesis activity against human hepatocellular carcinoma. We uncovered that two major signaling pathways were involved in deguelin-mediated neovascularization inhibition. First, deguelin decreased the activity of angiogenesis in endothelial cells via suppressing VEGF-induced VEGFR2 activation (Figures 2–4). Second, deguelin down-regulated the proangiogenic characteristics in hepatocellular carcinoma cells by inhibiting the secretion of VEGF through the inhibition of HGF-c-Met signaling pathway (Figures 4–5).

Deguelin exhibited significant anti-tumorigenesis and anti-proliferative activity in various types of human cancer both *in vitro* and *in vivo*. Deguelin induces cell apoptosis by blocking anti-apoptotic pathways, such as PI3K-Akt, IKK-IκBα-NF-κB and AMPK-mTOR-survivin pathways. Furthermore, deguelin inhibits tumor cell propagation and malignant transformation through p27-cyclinE-pRb-E2F1 cell cycle control and HIF-1α-VEGF anti-angiogenic pathways [26]. Previous studies have demonstrated that deguelin suppressed the growth of human tumors in nude mouse models via the down-regulation of tumor angiogenesis. Hu and colleagues showed that deguelin could inhibit the growth and lymphatic metastasis of human lung cancer through suppression of VEGFD pathway [32]. However, Lee and colleagues showed that deguelin suppressed the growth of human lung cancer cells by down-regulation of HIF1α-mediated VEGFA expression [33] in both normoxia and hypoxia conditions. Other studies also indicated that the anti-angiogenesis activity was one of the major mechanisms which involved in deguelin-mediated gastric cancer [34] and hepatocellular carcinoma [35] inhibition. However, the molecular mechanism has not yet been fully elucidated. The role of VEGF and VEGFR2 in modulating tumor-vascular growth and function is well established in both preclinical models and cancer patients [36]. Here, we demonstrated that deguelin inhibited VEGF-induced VEGFR2 activation, as well as the phosphorylation of downstream targets Akt and ERK1/2 in HUVECs (Figure 4). These results suggest that deguelin possesses a direct antimitotic effect on human endothelial cells, which may result in a reduction of cell proliferation, chemotactic motility, and tube formation.

Over-expression of c-Met or/and HGF has also been identified in the vast majority of solid tumors [37, 38]. Researchers have reported that the activation of HGF/c-Met signaling in cancer cells promoted VEGF biosynthesis in a PI3K signaling pathway-dependent manner [30, 31, 39]. Interestingly, HGF has been shown to not only increase VEGF production but to also act synergistically with VEGF to induce tube formation and angiogenesis [40–42]. Therefore, HGF/c-Met could be a potentially useful antiangiogenic target in human cancers. More importantly, the HGF/c-Met signaling is one of the most frequently dysregulated pathways in hepatocellular carcinoma [43], targeting of this pathway directly suppressed tumor growth and metastasis by a dual blockade of VEGFR2 and c-Met activation [44]. Here, results showed that deguelin inhibited the secretion of VEGF in both HepG2 and Hep3B cells in an HGF/c-Met signaling-depended manner (Figure 5). In agreement with the above results, the motility of endothelial cells triggered by VEGF in *ex vivo* and *in vivo* assays can be significantly inhibited by deguelin at low concentrations (Figure 2). Thus, the suppression of HGF/c-MET pathway in cancer cells may also contribute to the antitumor effects of deguelin through modulating the angiogenesis activity of human endothelial cells.

Receptor tyrosine kinase (RTK) pathways often regulate one another, and crosstalk between specific RTKs facilitates not only malignant tumor growth but also resistance to cancer therapy [45]. Disruption of either c-Met or VEGF only slowed tumor progression partially. More importantly, recent studies revealed that anti-VEGF or anti-angiogenesis cancer therapies promoted cancer metastasis [46–48]. Sennino and colleagues demonstrate that selectively inhibition of VEGF via the use of an anti-VEGF antibody increased invasion and metastasis in a c-Met–dependent manner. Strikingly, selective c-Met inhibition was sufficient to block these effects, providing a potential mechanism for and solution to overcome increased invasion in the face of anti-VEGF therapy [49]. These findings have potentially important translational implications, particularly for tumors that display significant co-expression or co-activation of c-Met and VEGFR in the malignant epithelial cell compartment. A promising strategy to overcome these limitations is to disrupt both RTK pathways together either at the ligand-receptor level or downstream. Importantly, in order to confirm the *in vivo* antitumor activity of deguelin, we developed the PDX model by using 3 different orthotopic hepatocellular carcinomas which exhibit similar clinical characteristics (Table 1). Data showed that deguelin effectively inhibited tumor growth without any cytotoxicity (Figure 6). This result implied that deguelin may develop as a novel clinical therapeutic agent against hepatocellular carcinoma in the future.

Overall our study indicated that deguelin exerted potent anti-angiogenesis activities via specifically targeting HGF-c-Met and VEGF-VEGFR pathways in cancer cells and endothelial cells, respectively. Deguelin is a potent angiogenesis inhibitor with the potential to become a useful agent in the clinical treatment of human hepatocellular carcinoma.

## MATERIALS AND METHODS

### Cell culture and reagents

Cells (HUVECs, HepG2, and Hep3B) from American Type Culture Collection (ATCC) were cultured at 37°C in a humidified incubator with 5% CO_2_ according to the ATCC protocols. Cells were cytogenetically tested and authenticated before being frozen. Each vial of frozen cells was thawed and maintained for 2 months (10 passages). MHCC97-H cells were purchased from Cell Bank of Chinese Academy of Sciences, Shanghai, China. Of note, HUVECs were cultured with M199, supplemented with 20% FBS, Endothelial cell growth supplement (30 µg/ml) and heparin (100 µg/ml), Human liver cancer cells HepG2, Hep3B, and MHCC97-H were grown in Eagle’s Minimum Essential Medium supplemented with 10% FBS and antibiotics. Deguelin and chemical reagents, including Tris, NaCl, SDS, and DMSO, for molecular biology and buffer preparation, were purchased from Sigma-Aldrich (St. Louis, MO, USA) Gefitinib and Linsitinib were purchased from Selleckchem (Houston, TX, USA).

### MTS assay

Human liver cancer cells were seeded (3 × 10^3^/well/100 μl) into 96-well plates and treated with various doses of deguelin for different time points as indicated. The viability was assessed by MTS assay (Promega, Madison, WI, USA) according to instructions provided.

### Anchorage-independent cell growth assay

The anchorage-independent cell growth assay was performed as described previously [50]. Briefly, cells were suspended (8,000 cells/ml) in 1 ml of 0.3% agar with Eagle’s basal medium containing 10% FBS, 1% antibiotics, and different concentrations of deguelin overlaid into six-well plates containing a 0.6% agar base. The cultures were maintained in a 37°C, 5% CO_2_ incubator for 1 to 2 weeks, and then colonies were counted under a microscope using the Image-Pro Plus software program (Media Cybernetics, Silver Spring, MD, USA).

### Flow cytometry

Flow cytometry was used to quantify cells in each phase of the cell cycle. Cells (2 × 10^5^) were seeded into 6 well plates and treated with various concentrations of deguelin for 24 h. Cells were harvested and washed with PBS for two times and then fixed in 70% ethanol overnight at 4°C. Cells were counterstained in the dark with 50 μg/ml propidium iodide and 0.1% ribonuclease A (RNase A) in 400 μl of PBS at 25°C for 30 min. Stained cells were assayed and quantified using a FACSort Flow Cytometer (BD, San Jose, CA, USA).

### The motility assay

The assay to measure motility of HUVECs was based on “scratch” wounding a confluent monolayer. Cells were seeded onto 0.1% gelatin-coated six-well plates in complete medium. After 24 h, HUVECs were starved to inactivate cell proliferation when a confluent monolayer was formed. The cells were scratch wounded using the tip of a universal 10 μl pipette tip. ECGM containing 0.5% FBS was added with or without 10 ng/mL VEGF and different concentrations of deguelin. After 12 h incubation, the cells were rinsed with PBS and randomly chosen fields were photographed under a light microscope. The number of migrated cells was counted.

### *In vitro* capillary tube formation

HUVECs were pretreated with various dilutions of deguelin for 30 min and then seeded in 24 well plates coated with Matrigel (BD Biosciences, San Jose, CA, USA). After 6–8 h, tubular structures of endothelial cells were photographed using an inverted microscope (Olympus, Tokyo, Japan). Three independent experiments were performed.

### Invasion assay

An invasion assay was conducted using a modified Boyden chamber and Matrigel (BD Bioscience)-coated polycarbonate nucleopore membranes (Corning; 8-μm pore size). Serum-free medium containing VEGF (10 ng/mL) was pipetted into the lower wells. HUVECs were trypsinized and suspended at a density of 1 × 10^5^ cells/100 μl in serum-free medium without VEGF. Cells were then pretreated with deguelin for 30 min and 100 μl of the cell suspension were loaded into the upper wells. The chamber was incubated in a 5% CO2 incubator at 37°C. After 8 h of incubation, the membrane was fixed and stained with crystal violet solution. Invasiveness was determined by counting the cells that passed through the filter.

### *In vivo* matrigel plug assay

Growth factor-reduced Matrigel (BD Biosciences) containing 80 ng VEGF and 20 units of heparin with or without deguelin were implanted subcutaneously into C57/BL/6 mice. After 7 days, the mice were euthanized and Matrigel plugs were removed. H&E staining was performed and functional microvessels were quantified manually using a microscope to identify the formation of new microvessels.

### VEGFR2 inhibition assay

The *in vitro* VEGFR2 inhibition assay was performed using the HTScan^®^ VEGF Receptor 2 Kinase Assay Kit (#7788, Cell Signaling Technology) according to manufacturers’ standard procedures.

### Protein preparation and western blotting

Protein samples were extracted with RIPA buffer (10 mM Tris-Cl (pH 8.0), 1 mM EDTA, 0.5 mM EGTA, 1% Triton X-100, 0.1% sodium deoxycholate, 0.1% SDS, 140 mM NaCl). Protein concentration was determined using the BCA Assay Reagent (Pierce, Rockford, IL, USA). For immunoblotting, proteins (30 μg) were detected with specific antibodies and an HRP-conjugated secondary antibody. Primary antibodies were used for immunoblotting: p-VEGFR (#2478, 1:1000), VEGFR (#9698, 1:2000), p-Akt (#4060, 1:1000), panAkt (#4691, 1:2000), p-ERK1/2 (#8544, 1:2000), ERK1/2 (#4695, 1:2000), p-EGFR(#3777, 1:1000), EGFR (##4267, 1:2000), p-IGF1Rβ (#28897, 1:1000), IGF1Rβ (#9750), p-c-Met (#3077, 1:1000) and c-Met (#8198, 1:2000) from Cell Signaling Technology; β-Actin (A5316, 1:10000) from Sigma-Aldrich. Secondary antibodies anti-rabbit IgG HRP (#7074, 1:10000) and anti-mouse IgG HRP (#7076, 1:10000) were purchased from Cell Signaling Technology. Antibody conjugates were visualized by chemiluminescence (ECL, Thermo Scientific, USA).

### Lentiviral infection

Lentivirus plasmids containing *pLKO.1-shc-Met* (TRCN0000040043, TRCN0000040044)*, pLKO.1-shEGFR* (TRCN0000039633, TRCN0000039636) and *pLKO.1-shIGF1Rβ* (TRCN0000000422, TRCN0000000426) were purchased from GE Dharmacon. The *c-Met* overexpression plasmid (RC217003) was purchased from OriGene Technologies, Inc. (Rockville, MD, USA). The *pLKO.1-shGFP* (Addgene plasmid #30323), the lentiviral packaging plasmid *psPAX2* (Addgene plasmid #12260) and the envelope plasmid *pMD2.G* (Addgene plasmid #12259) were available on Addgene (Cambridge, MA). The generation of gene stable knocking down cell lines was performed as described previously [51]. Briefly, *pLKO.1-sh-GFP* or *pLKO.1-shRNA* lentivirus plasmids were co-transfected into 293T cells with *psPAX2* and *pMD2-G*. Viral supernatant fractions were collected at 48 hours after transfection and filtered through a 0.45 μm filter followed by infection into cells together with 10 μg/mL polybrene. At 16 hours after infection, the medium was replaced with fresh medium containing 1 μg/ml puromycin and cells were incubated for another 6 days.

### VEGF ELISA assay

Human VEGF was measured by ELISA kit (R&D Systems, Minneapolis, MN, USA), according to the manufacturer’s instructions. Each sample was analyzed in duplicate.

### *In vivo* tumor growth assay

All the experimentation for animals was approved by the Animal Ethics Committee of Central South University, following the Guidelines of Animal Handling and Care in Medical Research from Hunan Province, China. HepG2 cells (3 × 10^6^) in 100 μl RPMI-1640 medium were inoculated s.c. into the right flank of 6-week-old female athymic nude mice. Nude mice (*n* = 5) were randomly divided into groups when tumor volume reached 50 to 100 mm^3^. The dosage of deguelin was 4 mg/kg and was administrated every three days by intraperitoneal injection, whereas control mice were administered vehicle. The body weight of each mouse was recorded and tumor volume was determined by vernier caliper twice a week. Volume was calculated following the formula of A × B^2^ × 0.5, wherein A is the longest diameter of tumor, B is the shortest diameter and B^2^ is B squared. Mice were monitored until day 34 and at that time mice were euthanized and tumors extracted.

### Establishment of PDX model

Human tissue collection and use protocols were approved by the ethics committee of Central South University, Changsha, Hunan, China. Hepatocellular carcinoma samples were obtained from 3 patients who were informed and provided written consent. Patients were treated with surgery at the Third Xiangya Hospital of Central South University (Changsha, Hunan, China). Pathologic and clinical data were entered and maintained in our prospective database.

The method was performed as described previously [52]. Briefly, fresh tumor tissue fragments were collected and transferred into an ice-cold serum-free RPMI-1640 medium with antibiotics. Within 2 h of surgical resection, tumor tissues were trimmed, cut into 3–5 mm sizes and implanted subcutaneously in anesthetized 6 to 8 week old female C.B-17 severe combined immunodeficient (SCID) mice (Vital River Laboratories Co., Ltd., Beijing, China). Once mass formation reached about 1500 mm^3^, mice of this first generation of xenografts (named P1) were sacrificed and the tumors were passaged and expanded for 2 more generations (named P2 and P3). When P3 tumors reached an average volume of 50–100 mm^3^, mice were divided into 2 groups (*n* = 5 mice per group) and treated with vehicle, or 4 mg/kg deguelin, respectively, every 3 days by intraperitoneal (i.p.) injection. Tumor volume and body weight were determined by vernier caliper twice a week.

### Immunohistochemical analysis

A Vectastain Elite ABC Kit (Vector Laboratories; Burlingame, CA) was used for immunohistochemical staining according to the recommended protocol. Briefly, the slide was baked at 60°C for 2 h, deparaffinized, and rehydrated. To expose antigens, the slide was unmasked by submersion into boiling sodium citrate buffer (10 mM, pH 6.0) for 10 min, and then treated with 3% H_2_O_2_ for 10 min. The slide was blocked with 50% goat serum albumin in 1×PBS in a humidified chamber for 1 h at room temperature and then with a first antibody (1:100 dilution in 50% goat serum with PBS) at 4°C in a humidified chamber overnight. The slide was washed and hybridized with the secondary antibody from Vector Laboratories (Burlingame, CA) (anti-rabbit 1:200) for 1 h at room temperature. Slides were stained using the Vectastain Elite ABC kit.

### Statistical analysis

Standard statistical methods were performed using Statistics Package for Social Science (SPSS) software (version 13.0; SPSS, Chicago, IL, USA). All data are presented as mean values ± S.D. as indicated and analyzed using the Student’s *t*-test or ANOVA. A *p* value < 0.05 was considered statistically significant.

## SUPPLEMENTARY MATERIALS FIGURES



## Abbreviations

HCC: hepatocellular carcinoma
VEGF: vascular endothelial growth factor
HUVECs: human umbilical vascular endothelial cells
HGF: hepatocyte growth factor
