# Supplementary material for: Deguelin suppresses angiogenesis in human hepatocellular carcinoma by targeting HGF-c-Met pathway
Source: Oncotarget. 2017 Oct 26;9(1):152–66. doi: 10.18632/oncotarget.22077 (PMC5787453; doi:10.18632/oncotarget.22077)
Supplement: Supplementary file 1 [file oncotarget-09-152-s001.pdf]

Deguelin suppresses angiogenesis in human hepatocellular carcinoma by targeting HGF-c-Met pathway

SUPPLEMENTARY MATERIALS

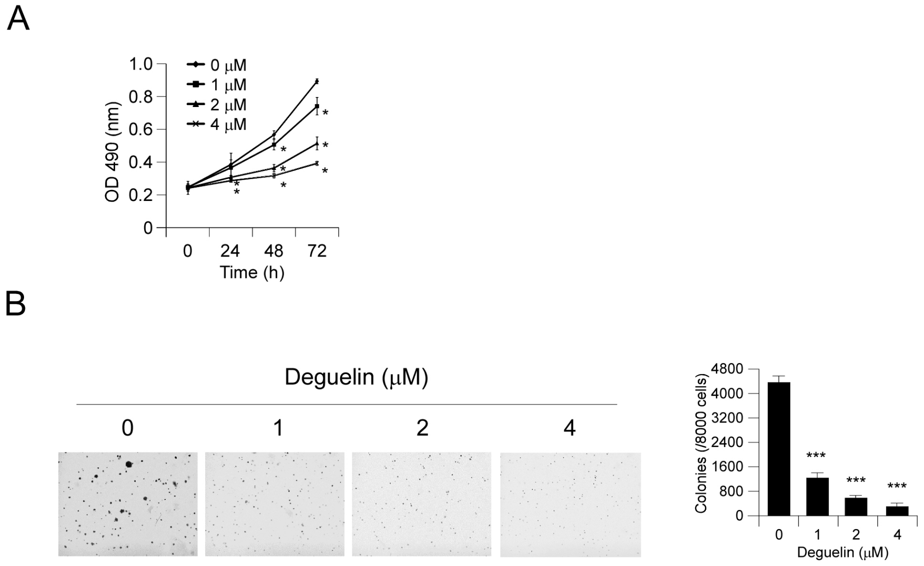

Supplementary Figure 1: Deguelin inhibits MHCC97-H cells growth *in vitro*. (**A**) Deguelin decreases cell growth. MHCC97-H cells were treated or not treated with deguelin as indicated, MTS assay was performed as described in Materials and Methods. The asterisk (*) indicates a significant ($p < 0.05$) decrease in cell proliferation by deguelin-treated cells. (**B**) Deguelin attenuates MHCC97-H cells anchorage-independent cell growth. Soft agar assay was performed as described in Materials and Methods. The asterisk (***) indicates a significant ($p < 0.001$) decrease in colony formation by deguelin-treated cells.

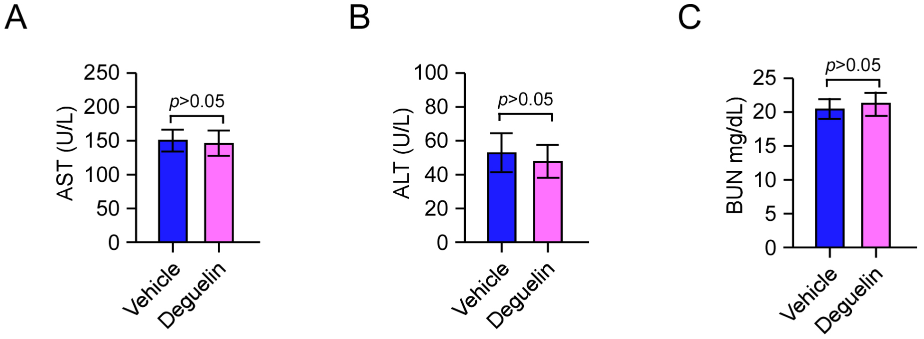

Supplementary Figure 2: Toxicity analysis for treatment with vehicle/deguelin in mice bearing HepG2 xenografts. Blood analysis of mice after treatment for 34 days. (**A**) AST, Aspartate Aminotransferase, (**B**) ALT, Alanine Aminotransferase, (**C**) BUN, Blood Urea Nitrogen.

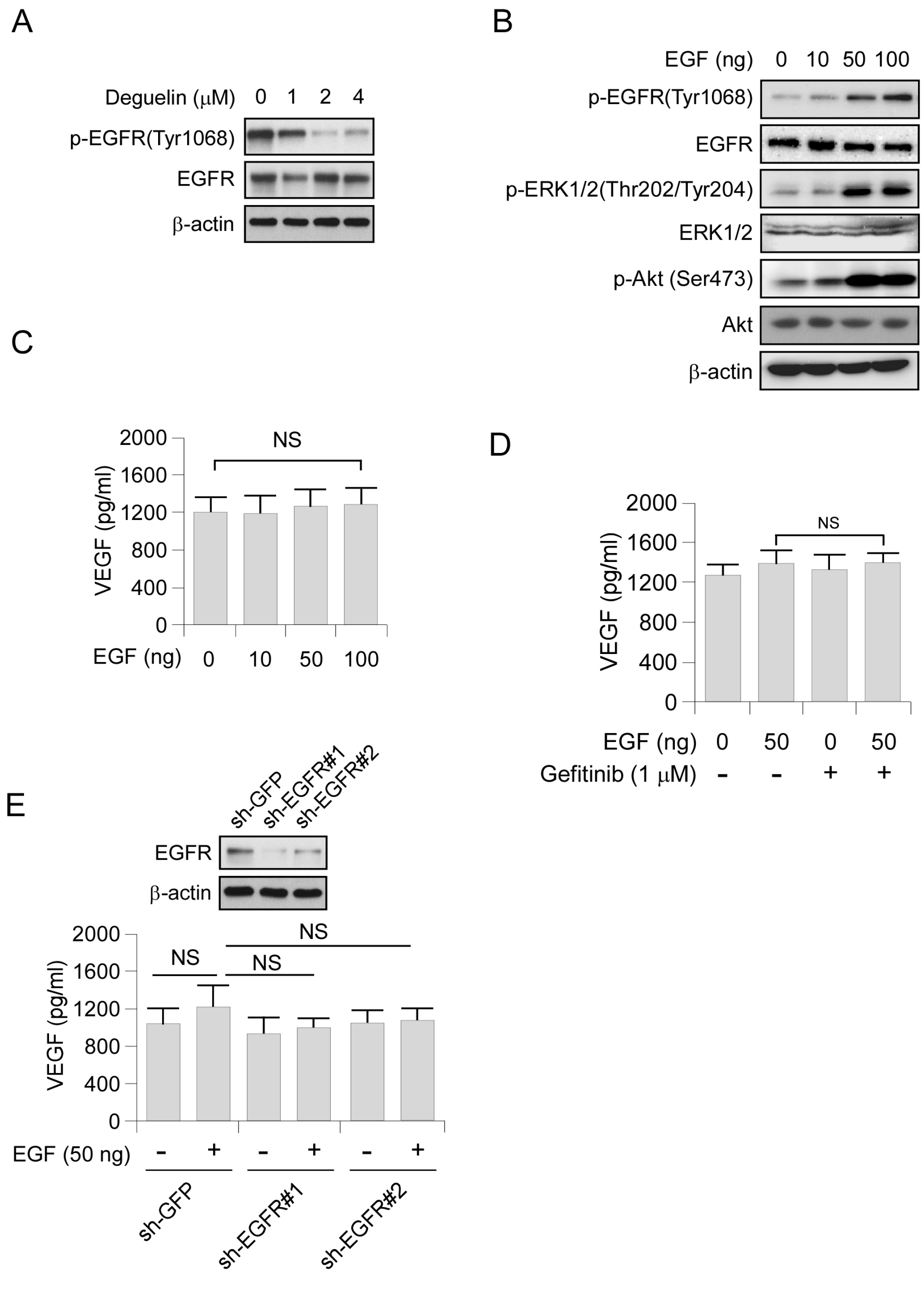

Supplementary Figure 3: The EGF-EGFR signaling pathway is not required for deguelin-mediated VEGF production suppression. (**A**) Deguelin inhibits EGFR activity. HepG2 cells were treated with different concentrations of deguelin for 12 h. Western blot was performed to analyze the phosphorylation of EGFR. (**B**) EGF activates EGFR signaling pathway. HepG2 cells were treated with various dosages of EGF for 12 h, whole cell lysates were subject to Western blot analysis. (**C**) EGF stimulation has no obvious effect on VEGF secretion. HepG2 cells were treated with different concentrations of EGF for 12 h. Cell culture media was harvested and VEGF level was measured by ELISA assay. (**D**) The effect of gefitinib on VEGF secretion. HepG2 cells were treated with EGF/gefitinib for 12 h as indicated. Cell culture media was harvested and VEGF level was measured by ELISA assay. (**E**) The effect of Knocking down of EGFR on EGF-induced VEGF secretion. The EGFR expression was tested by Western blot (top). The sh-GFP and sh-EGFR cells were treated with or without EGF for 12 h, cell culture media was subjected to ELISA assay.

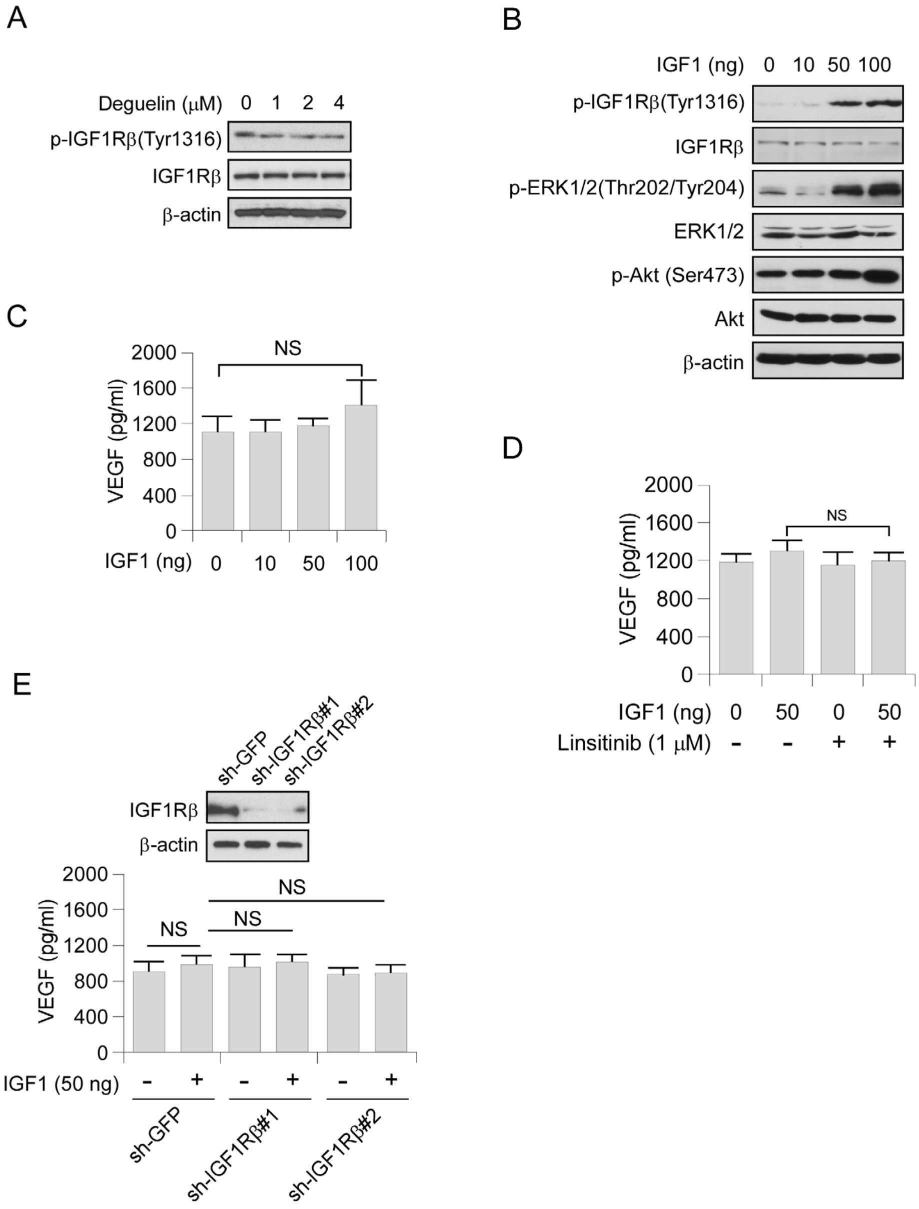

Supplementary Figure 4: The IGF1-IGF1R signaling pathway is not required for deguelin-mediated VEGF production suppression. (**A**) Effect of deguelin on IGF1Rβ activity. HepG2 cells were treated with different concentrations of deguelin for 12 h. Western blot was performed to analyze the phosphorylation of IGF1Rβ. (**B**) IGF1 activates IGF1R signaling pathway. HepG2 cells were treated with various dosages of IGF1 for 12 h, whole cell lysates were subject to Western blot analysis. (**C**) IGF1 stimulation has no obvious effect on VEGF secretion. HepG2 cells were treated with different concentrations of IGF1 for 12 h. Cell culture media was harvested and VEGF level was measured by ELISA assay. (**D**) The effect of linsitinib on VEGF secretion. HepG2 cells were treated with IGF1/ linsitinib for 12 h as indicated. Cell culture media was harvested and VEGF level was measured by ELISA assay. (**E**) The effect of Knocking down of IGF1Rβ on IGF1-induced VEGF secretion. The IGF1Rβ expression was tested by Western blot (top). The sh-GFP and sh- IGF1Rβ cells were treated with or without IGF1 for 12 h, cell culture media was subjected to ELISA assay.
